# Arthroscopy Association of Canada Position Statement on Opioid Prescription After Arthroscopic Surgery

**DOI:** 10.1177/23259671231214700

**Published:** 2023-12-22

**Authors:** Nicholas Nucci, Ryan Degen, Seper Ekhtiari, Aaron Gazendam, Olufemi R. Ayeni, Nolan Horner, Ivan Wong, Jarret Woodmass, John Grant, Brendan Sheehan, Michael Pickell, Michaela Kopka, Moin Khan, Ryan Martin, Allison Tucker, Mark Sommerfeldt, Eva Gusnowski, Alexis Rousseau-Saine, Marie-Eve Lebel, Jillian Karpyshyn, Bogdan Matache, Michael Carroll, Rachael Da Cunha, Adam Kwapisz, R. Kyle Martin

**Affiliations:** University of Ottawa, Ottawa, Ontario, Canada; Western University, London, Ontario, Canada; McMaster University, Hamilton, Ontario, Canada; McMaster University, Hamilton, Ontario, Canada; McMaster University, Hamilton, Ontario, Canada; Genesis Orthopaedics & Sports Medicine, Chicago, Illinois, USA; Dalhousie University, Halifax, Nova Scotia, Canada; University of Manitoba, Winnipeg, Manitoba, Canada; University of Michigan, Ann Arbor, Michigan, USA; Saint John Orthopaedics, Saint John, New Brunswick, Canada; University of Ottawa, Ottawa, Ontario, Canada; Banff Sport Medicine, Canmore, Alberta, Canada; McMaster University, Hamilton, Ontario, Canada; University of Calgary, Calgary Alberta, Canada; Nova Scotia Health Authority, Halifax Nova Scotia, Canada; University of Alberta, Edmonton Alberta, Canada; Saint John Orthopaedics, Saint John, New Brunswick, Canada; University of Montreal, Montreal, Quebec, Canada; Western University, London, Ontario, Canada; University of Alberta, Edmonton Alberta, Canada; Laval University, Quebec City Quebec, Canada; Queen Elizabeth Hospital, Charlottetown, Prince Edward Island, Canada; Queen’s University, Kingston Ontario, Canada; University of Manitoba, Winnipeg, Manitoba, Canada; University of Minnesota, Minneapolis, Minnesota, USA; Investigation performed at McMaster University, Hamilton, Ontario, Canada

**Keywords:** arthroscopy, opioids, postoperative, reduction

## Abstract

**Background:**

Despite the ongoing opioid epidemic, most patients are still prescribed a significant number of opioid medications for pain management after arthroscopic surgery. There is a need for consensus among orthopaedic surgeons and solutions to aid providers in analgesic strategies that reduce the use of opioid pain medications.

**Purpose:**

This position statement was developed with a comprehensive systematic review and meta-analysis of exclusively randomized controlled trials (RCTs) to synthesize the best available evidence for managing acute postoperative pain after arthroscopic surgery.

**Study Design:**

Position statement.

**Methods:**

The Embase, MEDLINE, PubMed, Scopus, and Web of Science databases were searched from inception until August 10, 2022. Keywords included *arthroscopy*, *opioids*, *analgesia*, and *pain*, and associated variations. We included exclusively RCTs on adult patients to gather the best available evidence for managing acute postoperative pain after arthroscopic surgery. Patient characteristics, pain, and opioid data were extracted, data were analyzed, and trial bias was evaluated.

**Results:**

A total of 21 RCTs were identified related to the prescription of opioid-sparing pain medication after arthroscopic surgery. The following recommendations regarding noninvasive, postoperative pain management strategies were made: (1) multimodal oral nonopioid analgesic regimens—including at least 1 of acetaminophen—a nonsteroidal anti-inflammatory drug—can significantly reduce opioid consumption with no change in pain scores; (2) cryotherapy is likely to help with pain management, although the evidence on the optimal method of application (continuous-flow vs ice pack application) is unclear; (3) and (4) limited RCT evidence supports the efficacy of transcutaneous electrical nerve stimulation and relaxation exercises in reducing opioid consumption after arthroscopy; and (5) limited RCT evidence exists against the efficacy of transdermal lidocaine patches in reducing opioid consumption.

**Conclusion:**

A range of nonopioid strategies exist that can reduce postarthroscopic procedural opioid consumption with equivalent vocal pain outcomes. Optimal strategies include multimodal analgesia with education and restricted/reduced opioid prescription.

Arthroscopic techniques in orthopaedic surgery aim to utilize minimally invasive strategies to reduce the surgical risks of open approaches. This includes reducing postoperative pain. However, most patients are still prescribed a significant number of opioid medications for pain management after arthroscopic surgery.^30^

The opioid epidemic is ongoing, and consumption continues to rise.^22^ Increasing opioid-related mortality data spans all racial and ethnic backgrounds, stemming from both prescription and illicit opioid use.^18^ More than half of all opioid deaths are related to prescription opioids.^8^ Orthopaedic surgeons remain a significant contributor to the prescription of opioid medications. Despite the majority of orthopaedic surgeons agreeing that the overprescription of opioids is of significant concern, opioids rank among the highest prescribers of all medical specialties.^7,10^

Given the current magnitude of the issue, there is certainly an impetus for solutions to the opioid crisis. This begins with providing prescribers with guidance toward optimal opioid prescribing based on the best available evidence for the acute postoperative setting. There is a need for consensus among orthopaedic surgeons and solutions to aid providers in analgesic strategies that reduce the use of opioid pain medications.

The current position statement from the Arthroscopy Association of Canada provides the most up-to-date, evidence-informed, nonprocedural postoperative pain management techniques being utilized in orthopaedic arthroscopic randomized controlled trials (RCTs) to reduce opioid consumption. The goal was to assist in decision-making regarding postoperative pain control strategies to reduce opioid prescription and limit the potential long-term sequelae with their continued use.

## Methods

This position statement was developed with a comprehensive systematic review and meta-analysis of exclusively RCTs to provide the best available evidence for managing acute postoperative pain after arthroscopic surgery. The Embase, MEDLINE, PubMed, Scopus, and Web of Science databases were searched from inception until August 10, 2022. Keywords included *arthroscopy*, *opioids*, *analgesia*, and *pain*, and associated variations. An independent screening process was completed for both title and abstract screening in addition to full-text review. RCTs were extracted, data were analyzed, and study bias was evaluated. This informed the data and recommendations of the current position statement.

Many postoperative techniques exist to reduce the prescription of opioids in the postoperative period. The quality of evidence and strength of recommendations were rated according to the Strength of Recommendations Taxonomy (SORT) guidelines^5^ (Table 1).

**Table 1:** The SORT Guidelines^5,*a*^

Strength of Evidence	Definition
A	Recommendations based on consistent and good quality patient-oriented evidence
B	Recommendations based on inconsistent or limited quality patient-oriented evidence
C	Recommendations based on consensus, usual practice, opinion, disease-oriented evidence, and case series for studies of diagnosis, treatment, prevention, or screening

a SORT, Strength of Recommendations Taxonomy.

## Results

A total of 21 RCTs^1-4,9,11-13,15,16,19,20,23-29,33,34^ were identified as related to the prescription of opioid-sparing pain medication after arthroscopic surgery. A review of available data provided the following recommendations regarding 5 noninvasive postoperative pain management strategies: (1) oral nonopioid analgesics; (2) cryotherapy; (3) transcutaneous electrical nerve stimulation (TENS); (4) postoperative relaxation exercises; and (5) transdermal lidocaine patches. Findings were compiled in a visual aid available separately as supplemental material.^6^

### Oral Nonopioid Analgesics

- *Recommendation*. Multimodal oral nonopioid analgesic regimens, including at least 1 of acetaminophen—a nonsteroidal anti-inflammatory drugs (NSAIDs)—can significantly reduce opioid consumption, with no change in pain scores.^2-4,10-12,18,22-24,26,27^
- *Strength of Recommendation*. A
- *Recommendation*. Nonbenzodiazepine hypnotic sleep medications (eg, zolpidem) can significantly reduce opioid intake with no difference in pain scores.^27,29^
- *Strength of Recommendation*. B

### Cryotherapy

- *Recommendation*. Cryotherapy is likely to help with pain management, although the evidence on the optimal application method (continuous flow vs ice pack) is unclear.^1,15,34^
- *Strength of Recommendation*. B

### Transcutaneous Electrical Nerve Stimulation

- *Recommendation*. Limited RCT evidence supports the efficacy of TENS in reducing opioid consumption after arthroscopy.^20^
- *Strength of Recommendation*. B

### Postoperative Relaxation Exercises

- *Recommendation*. Limited RCT evidence supports the efficacy of relaxation exercises in reducing opioid consumption after arthroscopy.^33^
- *Strength of Recommendation*. B

### Transdermal Lidocaine Patches

- *Recommendation*. Limited RCT evidence exists against the efficacy of transdermal lidocaine patches in reducing opioid consumption.^16^
- *Strength of Recommendation*. B

The characteristics of the reviewed RCTs are summarized in Table 2.

**Table 2:** Characteristics of the Included Studies^*a*^

Lead Author (Year)	LOE	Arthroscopic Surgery Type	Sample Size	Intervention	Control
Mahure^20^ (2017)	2	RTCR	37	Active TENS	Placebo TENS
Jildeh^12^ (2022)	1	RTCR	40	Multimodal analgesia	Opioid analgesia
Guo^9^ (2022)	1	Knee	126	Imrecoxib	Celecoxib
Sivasundaram^25^ (2021)	1	RTCR	39	Oral ketorolac + percocet	Percocet
Bloom^3^ (2021)	1	Hip	80	Acetaminophen + oxycodone	Acetaminophen
Hartwell^11^ (2020)	1	Knee partial meniscectomy	95	Printed oxycodone script	Faxed oxycodone script
Tashjian^27^ (2006)	1	Knee	67	Zolpidem 10 mg + opioid analgesia	(1) Opioid analgesia, (2) placebo + opioid
Tangtiphaiboontana^26^ (2021)	1	RTCR	101	Ibuprofen	Placebo
Tompkins^29^ (2011)	1	ACLR	29	Zolpidem	Placebo
Weekes^33^ (2021)	1	RTCR	146	Relaxation exercises + opioid analgesia	Opioid analgesia
Jildeh^13^ (2021)	1	Labral	48	Nonopioid multimodal analgesia	Percocet
Thompson^28^ (2021)	1	Shoulder capsulolabral repair	80	Ibuprofen	Percocet
Woolf^34^ (2008)	1	Knee	53	Continuous cryotherapy	Ice pack
Binning^2^ (2007)	1	Knee meniscectomy	94	Nimesulide	(1) Naproxen, (2) placebo
Chelly^4^ (2007)	1	Knee	439	Rofecoxib	(1) Hydrocodone/acetaminophen, (2) placebo
Barber^1^ (1998)	1	ACLR	100	Continuous-flow cold therapy	No continuous-flow cold therapy
Singh^24^ (2021)	2	RTCR	57	Postop acetaminophen PRN + oxycodone PRN	(1) Oxycodone PRN, (2) postop acetaminophen PRN + oxycodone PRN
Lee^16^ (2022)	2	RTCR	56	Lidocaine patch	Standard care
Liu^19^ (2021)	1	Knee	232	Celecoxib	Oxycodone-paracetamol
Kijkunasathian^15^ (2017)	1	ACLR	38	Modified Robert Jones bandage	Intermittent cold pack
Gazendam^23^ (2022)^2^	1	Shoulder or knee	193	Opioid-sparing protocol	Standard care

a ACLR, anterior cruciate ligament reconstruction, LOE, level of evidence; postop, postoperative; PRN, as needed; RTCR, rotator cuff repair; TENS, transcutaneous electrical nerve stimulation.

## Discussion

### Oral Analgesics

We identified 15 RCTs^2-4,9,11-13,19,23-29^ that utilized oral analgesics to reduce opioid consumption. Strategies varied but always included the use of acetaminophen or an NSAID in their analgesic regimen. These prescriptions may have been in replacement or conjunction with opioids.

Adjunct medications were broad, including at least 1 of the following: acetaminophen, aspirin, celecoxib, gabapentin, ibuprofen, ketorolac, methocarbamol, naproxen, nimesulide, and rofecoxib. Some trials also included anticonvulsants, patient education, proton pump inhibitors, or steroids. No single NSAID has been shown to be superior to others, and newer NSAIDs—such as nimesulide and imrecoxib—have demonstrated similar efficacy to the more commonly used NSAIDs. Overall, regardless of the exact regimen used, acceptable pain management was achieved across the vast majority of studies, with many studies showing reductions in opioid consumption in the postoperative period. Nonbenzodiazepine sleeping aids (Zolpidem) were also investigated and found to achieve satisfactory pain relief with significant reductions in opioid use compared with standard regimens. Although Zolpidem is a nonbenzodiazepine sleep aid as such we advise caution with its use. Zolpidem demonstrates some evidence for opioid reduction after arthroscopic surgery but may also induce pharmacodependence and has substance abuse potential.^31,32^ Of note, those with a baseline substance abuse history appear at greater risk for harmful use.^31^

We feel that, based on the data and our experience with RCTs on this topic, patient education is an important component of the efficacy of oral nonopioid analgesics. Often, patients are unclear about which nonprescription analgesics they can take to supplement their opioid medications and which classes of medications interact with one another. As demonstrated here, in the context of an RCT, where the patient is being actively monitored and encouraged to take the medication of interest, patients do achieve substantial benefits compared with standard opioid-focused medication regimens. While we can expect a Hawthorne effect in the context of a clinical study, we propose that the added information that the patient receives in the enrollment process and the legitimizing effect of having a clinician endorse (or prescribe) the use of nonopioid oral analgesics also play a role in the demonstrated benefit of these medications.^21^

### Cryotherapy

Cryotherapy was utilized in 3 RCTs^1,15,34^ in a variety of methods, including continuous flow cold therapy, ice, and cold packs. Cryotherapy does demonstrate some benefits in reducing short-term opioid consumption and pain scores. However, the results were mixed. The best evidence exists for continuous cold flow-reducing opioids in the short-term postoperative period.^1^ There appeared to be no significant difference in opioid consumption and postoperative pain when comparing continuous flow to ice packs and intermittent cold packs to Robert Jones bandages. However, ice/cold packs and Jones bandages have yet to be compared with noncryotherapy controls in randomized trials.^15,34^

We support the use of cryotherapy—preferential through a continuous cold flow system—to reduce postoperative pain and opioid consumption. In addition to the aforementioned results, this low-risk adjunct had the benefit of increased range of motion and exercise adherence postoperatively.^1^

### Transcutaneous Electrical Nerve Stimulation

TENS has been utilized with success in the short term after arthroscopic rotator cuff repair. Active therapy reduced opioid consumption and improved pain scores when compared with controls.^20^ As a low-cost and accessible treatment adjunct, we support the use of TENS postoperatively to reduce opioid consumption. Risks are minimal, and burns at the electrode sites have been reported very rarely. However, TENS should be provided by a professional with experience in the use of the modality.^14^

### Relaxation Exercises

Relaxation exercises demonstrate a low-cost, accessible adjunct to postoperative care. Weekes et al^33^ found that after rotator cuff arthroscopic repair, there was no significant difference in pain scores. Still, there was a significant decrease in opioid consumption in favor of the relaxation exercise group versus the standard care group. Relaxation exercises are an easily implemented method that increases the patient's perception of control and can decrease anxiety.^33^ Therefore, we support their use in the postoperative period.

### Transdermal Patches

Transdermal lidocaine patches are not effective in reducing pain and opioid consumption after arthroscopy rotator cuff repair. In fact, lidocaine patch users report worse short-term satisfaction with their pain management compared with controls.^16^ For these reasons, we do not recommend their use in the postoperative period.

### Suggested Opioid Reduction Protocol

Our suggested opioid reduction protocol includes a multimodal, evidence-based, and harm-minimizing approach. In addition to oral analgesics, a range of other modalities have some evidence to support their use, and we recommend that these be considered adjuncts, particularly if they are available at low cost to patients and pose very little risk of harm. The protocol from Gazendam et al is shared in Table 3 as one that is relatively simple to implement. The opioid-reduced postoperative pain protocol was created using a multidisciplinary approach and implemented with success in patients after arthroscopic knee and shoulder surgery in a high-quality RCT by the NO PAin Investigators.^23^ In their protocol, which was supplemented with a patient education infographic, all medications were prescribed on an as-needed basis. However, patients were encouraged to use both naproxen and acetaminophen in the first 7 days postoperatively, even when experiencing mild pain^23^ (Table 3).

**Table 3:** Sample Postoperative Opioid-Sparing Protocol^23^

Medication	Route and Frequency	Dispensing
Naproxen	Oral, twice daily, as needed	60 × 250–mg tablets
Pantoprazole	Oral, daily, while consuming naproxen	30 × 40–mg tablets
Acetaminophen	Oral, 4 times daily, as needed	100 × 500–mg tablets
Hydromorphone^*a*,*b*,*c*^	Oral, every 4 hours, as needed	10 × 1–mg tablets

a Given as a separate prescription from the nonopioid medications.

b 5-mg morphine in the case of allergies.

c Patients were instructed to take the opioid only when the nonopioid protocol provided inadequate analgesia.

### Strengths and Limitations

The strengths of our position statement include the high-quality methodologic design of the source papers. This position statement demonstrates the best available evidence, to the authors’ knowledge, at the time of publication submission. The data presented and characteristics highlighted in Table 2 demonstrate a variety of patient populations, injuries, and affected joints on which procedures were performed. As this spanned a variety of arthroscopy procedures with a large patient population, our recommendations are generalizable to many orthopaedics patients after arthroscopy.

The primary weaknesses of our recommendations are related to the source data. The heterogeneity of nonopioid oral analgesic protocols, inconsistent reporting, and low number of studies for certain analyzed adjuncts all limit the strength of conclusions for our above recommendations.

Although many risks were not explicitly discussed in this position statement, as with any medication prescription, proper counseling on risks for both opioid and nonopioid medications alike must be performed. This is of particular concern with Zolpidem use, which may induce pharmacodependence and has the potential for abuse.^31,32^ Risks, including the ones mentioned, and specifics related to the prescribed medication, should be discussed with the patient before use.

### Future Research

Our position statement and associated systematic review/meta-analysis highlight some areas for future research. Although a strong body of literature exists for various multimodal nonopioid analgesic strategies, meta-analysis could be improved with future RCTs evaluating data at multiple meaningful time points and making supplementary data available for analysis. Studies examining postoperative adjuncts to lower opioid consumption go beyond multimodal oral analgesic methods, which limits our ability to meaningfully alter clinical practice. A concerted effort to perform high-quality trials examining these interventions should be performed in the future.

## Conclusion

As demonstrated by our review, there is a range of nonopioid strategies that can reduce postarthroscopic procedural opioid consumption with equivalent pain outcomes. Optimal strategies include multimodal analgesia with education and restricted/reduced opioid prescription. Additional oral analgesia options can consist of Zolpidem, which demonstrated decreased postoperative opioid consumption and pain scores.^27^ Imrecoxib, a novel cyclooxygenase-2 inhibitor, can also be utilized, as it has been demonstrated to be noninferior to celecoxib in reducing pain and opioid consumption.^9^ This further demonstrates the array of analgesics that are suitable for postoperative opioid-sparing protocols.

Some limited evidence supports the use of TENS, cryotherapy, and relaxation exercises for reducing opioid consumption after arthroscopy. TENS data, although limited in arthroscopy has been shown in an RCT meta-analysis of arthroplasty patients to reduce opioid consumption and pain scores postoperatively.^17^ This position statement may strengthen the small data set supporting its use in arthroscopy. Cryotherapy and relaxation exercises both demonstrate inexpensive strategies for reducing opioid consumption with low side-effect profiles and, therefore, should also be considered.

## Authors

Nicholas Nucci, MD (University of Ottawa, Ottawa, Ontario, Canada); Ryan Degen, MD (Western University, London, Ontario, Canada); Seper Ekhtiari, MD (McMaster University, Hamilton, Ontario, Canada); Aaron Gazendam, MD (McMaster University, Hamilton, Ontario, Canada); Olufemi R. Ayeni, MD (McMaster University, Hamilton, Ontario, Canada); Nolan Horner, MD (Genesis Orthopaedics & Sports Medicine, Chicago, Illinois, USA); Ivan Wong, MD, (Dalhousie University, Halifax, Nova Scotia, Canada); Jarret Woodmass, MD (University of Manitoba, Winnipeg, Manitoba, Canada); John Grant, MD (University of Michigan, Ann Arbor, Michigan, USA); Brendan Sheehan, MD (Saint John Orthopaedics, Saint John, New Brunswick, Canada); Michael Pickell, MD (University of Ottawa, Ottawa, Ontario, Canada); Michaela Kopka, MD (Banff Sport Medicine, Canmore, Alberta, Canada); Moin Khan, MD (McMaster University, Hamilton, Ontario, Canada); Ryan Martin, MD (University of Calgary, Calgary Alberta, Canada); Allison Tucker, MD (Nova Scotia Health Authority, Halifax Nova Scotia, Canada); Mark Sommerfeldt, MD (University of Alberta, Edmonton Alberta, Canada); Eva Gusnowski, MD (Saint John Orthopaedics, Saint John, New Brunswick, Canada); Alexis Rousseau-Saine, MD (University of Montreal, Montreal, Quebec, Canada); Marie-Eve Lebel, MD (Western University, London, Ontario, Canada); Jillian Karpyshyn, MD (University of Alberta, Edmonton Alberta, Canada); Bogdan Matache, MD (Laval University, Quebec City Quebec, Canada); Michael Carroll, MD (Queen Elizabeth Hospital, Charlottetown, Prince Edward Island, Canada); Rachael Da Cunha, MD (Queen’s University, Kingston Ontario, Canada); Adam Kwapisz, MD (University of Manitoba, Winnipeg, Manitoba, Canada); and R. Kyle Martin, MD (University of Minnesota, Minneapolis, Minnesota, USA).

## Supplemental Material

sj-pdf-1-ojs-10.1177_23259671231214700 – Supplemental material for Arthroscopy Association of Canada Position Statement on Opioid Prescription After Arthroscopic Surgery Click here for additional data file. Supplemental material, sj-pdf-1-ojs-10.1177_23259671231214700 for Arthroscopy Association of Canada Position Statement on Opioid Prescription After Arthroscopic Surgery by Nicholas Nucci, Ryan Degen, Seper Ekhtiari, Aaron Gazendam, Olufemi R. Ayeni, Nolan Horner, Ivan Wong, Jarret Woodmass, John Grant, Brendan Sheehan, Michael Pickell, Michaela Kopka, Moin Khan, Ryan Martin, Allison Tucker, Mark Sommerfeldt, Eva Gusnowski, Alexis Rousseau-Saine, Marie-Eve Lebel, Jillian Karpyshyn, Bogdan Matache, Michael Carroll, Rachael Da Cunha, Adam Kwapisz and R. Kyle Martin in Orthopaedic Journal of Sports Medicine
