# Supplementary material for: Arthroscopy Association of Canada Position Statement on Opioid Prescription After Arthroscopic Surgery
Source: Orthop J Sports Med. 2023 Dec 22;11(12):23259671231214700. doi: 10.1177/23259671231214700 (PMC10748902; doi:10.1177/23259671231214700)
Supplement: sj-pdf-1-ojs-10.1177_23259671231214700 – Supplemental material for Arthroscopy Association of Canada Position Statement on Opioid Prescription After Arthroscopic Surgery [file sj-pdf-1-ojs-10.1177_23259671231214700.pdf]

Pain Management Strategies after Arthroscopic Surgery

EVIDENCE-BASED RECOMMENDATIONS

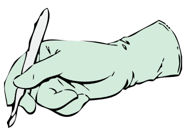	Intervention	Verdict
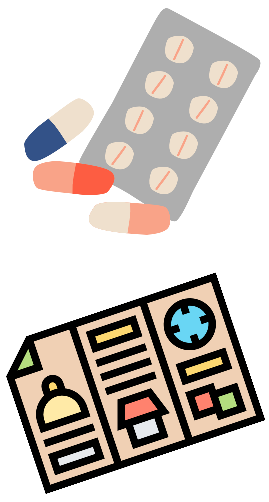	**Multimodal oral non-opioid analgesics and patient education**<br>• NSAIDs and acetaminophen reduce opioid consumption<br>• No specific NSAID superior<br>• Recommend combining with patient education	A, in favour
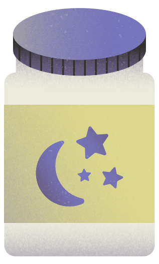	**Non-benzodiazepine sleeping aids**<br>• Limited evidence in favour of Zolpidem to reduce opioid intake	B, in favour
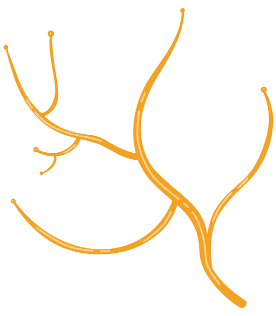	**Transcutaneous Electrical Nerve Stimulation (TENS)**<br>• Limited evidence in favour of TENS reducing opioid consumption	B, in favour
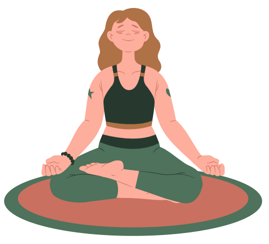	**Relaxation Exercises**<br>• Limited evidence in favour of relaxation exercises reducing opioid intake	B, in favour
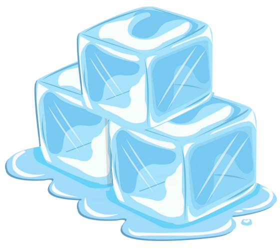	**Cryotherapy**<br>• Cryotherapy may help with pain management<br>• Unclear effect on opioid consumption<br>• Optimal method of delivery unclear	B, in favour
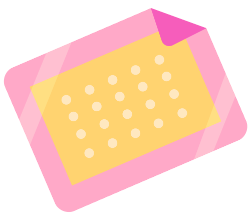	**Transdermal lidocaine**<br>• Limited evidence against transdermal lidocaine patches reducing opioid intake	B, against

Based on available evidence from randomized controlled trials
